# The Beit CURE Classification of Childhood Chronic Haematogenous Osteomyelitis—a guide to treatment

**DOI:** 10.1186/s13018-015-0282-9

**Published:** 2015-09-17

**Authors:** Andrew J. Stevenson, Henry Wynn Jones, Linda C. Chokotho, Verona LL Beckles, William J. Harrison

**Affiliations:** FRCS (T&O), North Bristol NHS Trust, 8 Mervyn Road, Bristol, BS7 9EL UK; FRCS (T&O), Wrightington, Wigan & Leigh NHS Foundation Trust, Wigan, UK; FCS (ECSA), Beit CURE International Hospital, Blantyre, Malawi; FRCS (T&O), North Middlesex University Hospital NHS Trust, London, UK; FRCS (T&O), Countess of Chester Hospital NHS Trust, Chester, UK

**Keywords:** Chronic, Osteomyelitis, Paediatric, Childhood, Haematogenous, Classification, Sequestrum, Sequestrectomy

## Abstract

**Background:**

The Beit CURE (BC) classification is a radiographic classification used in childhood chronic haematogenous osteomyelitis. The aim of this study is to assess correlation between this classification and the type and extent of treatment required.

**Methods:**

We present a retrospective series of 145 cases of childhood chronic haematogenous osteomyelitis classified using the BC classification. Variables measured include age, sex, bone involved, number of admissions, length of stay, type/number of operations and microbiology.

**Results:**

The most commonly affected bone was the tibia (46 %), followed by femur (26 %) and humerus (10 %). Bone defects were most common in the tibia. *Staphylococcus aureus* was the most commonly isolated organism.

Type B, sequestrum type, was the most common (88 %), followed by type C, sclerotic type, (7 %) and type A, Brodie’s abscess (5 %).

Types A and B1 had the shortest length of hospitalisation (11 days), type B4 had the longest (87 days). Types A and B1 had the fewest infection control operations. Type B4 had the greatest total number of operations.

**Conclusions:**

This study shows that the BC classification can guide surgical strategy and help predict length of inpatient treatment and number and type of procedures required.

## Background

Childhood chronic haematogenous osteomyelitis (CCHOM) is a major cause of disability and illness throughout the developing world and places significant burden on the resource-poor healthcare systems of these countries [1–3]. Patients have been shown to suffer weight loss and growth retardation [2], persistent sepsis [4, 5], joint/limb deformity and stiffness [2, 6] as well as social stigmatisation and exclusion from school. The incidence of osteomyelitis in the developing world is unknown, due to the paucity of data relating to the epidemiology of the disease. A study from Gambia showed that chronic osteomyelitis accounted for 15 % of surgical inpatient days and 5.7 % of admissions [7] In Burkina Faso, 5.3 % of hospital admissions were for chronic osteomyelitis [8]. In our institution (Beit CURE Malawi), it accounted for 7.6 % of inpatient days and 6.7 % of all paediatric operations [1].

The investigation of patients with CCHOM involves clinical evaluation, plain X-ray and blood tests (if available). Ultrasound, CT and MRI have been shown to be reliable modalities in both diagnosis and treatment planning [9]; however, in the resource-poor countries that this disease is most prevalent, these advanced diagnostics are often unavailable. Only one study of CCHOM in patients from resource-poor countries has used MRI, in which patients were expatriated to Germany for treatment [10]. This model is not transferable to the majority of CCHOM patients.

Consensus with regarding the basic surgical treatment of chronic osteomyelitis has largely been reached [1–6, 10–12]. The removal of dead bone/sequestrectomy, debridement, drainage of pus and soft tissue management are the mainstays of infection control. The specific role and mode of delivery of antibiotics in treatment remains a matter for debate. Some authors advocate antibiotics should be used routinely [2, 6, 10, 12–14], and others advocate that they should be used selectively or when patients are systemically unwell [1–4]. Opinion on the appropriate strategies for the treatment of bone defects also remains diverse and is often influenced by surgical experience and availability of resources [1, 17, 20–23].

There are numerous publications reporting on chronic osteomyelitis from resource-poor nations published in the last 25 years (Table 1). These studies are a largely retrospective case series. Out of these 18 studies, only five report exclusively on CCHOM with most studies reporting on osteomyelitis of mixed aetiology (i.e. post-traumatic, post-surgical), as well as including adults. Furthermore, only two of these studies used the same classification system, and 11 used no classification at all.

**Table 1:** Eighteen publications relating to CCHOM conducted in the last 26 years

Author [reference]	Year	Country	*N*	Type of OM	Age	Blood tests	XR used	MRI used	Microbiology reported	LoT	Classification used
Daoud [6]	1989	Algeria	34	CHOM	P	No	Yes	No	Yes	Yes	No
Meier [3]	1993	Nigeria	161	CHOM	P&A	No	Yes	No	Yes	No	Yes—own
Lauschke [2]	1994	Namibia	55	CHOM	P	Yes	Yes	No	Yes	No	Yes—own
Tekou [25]	2000	Togo	145	Mix	P	No	Yes	No	Yes	No	No
Bickler [7]	2000	Gambia	98	Mix	P	No	No	No	No	No	No
Solagberu [13]	2003	Nigeria	271	Mix	P&A	Yes	Yes	No	No	No	Yes—own
Kouame [24]	2005	Cote d’Ivoire	42	CHOM	P	Yes	Yes	No	Yes	No	No
Nacoulma [8]	2007	Burkina Faso	102	Mix	P&A	Yes	Yes	No	Yes	No	No
Erlap [17]	2007	Turkey	13	Mix	P&A	Yes	Yes	No	No	No	Cierny Mader
Museru [18]	2001	Tanzania	9	Mix	P	No	Yes	No	No	No	No
Akinyoola [19]	2008	Nigeria	47	Mix	P	Yes	Yes	No	Yes	Yes	No
Agaja [26]	2008	Nigeria	107	Mix	P&A	Yes	Yes	No	Yes	No	No
Liu [22]	2008	China	11	CHOM	P	Yes	Yes	No	No	Yes	No
Beckles [1]	2010	Malawi	167	CHOM	P	No	Yes	No	Yes	Yes	Beit CURE
Hung [13]	2010	Vietnam	376	Mix	P	No	Yes	No	Yes	No	Yes—own
Stoesser [16]	2013	Cambodia	81	Mix	P	Yes	Yes	No	Yes	Yes	No
Wirbel [10]	2014	Angola and Afghanistan	27	Mix	P	Yes	Yes	Yes	Yes	Yes	No
Ali [14]	2015	Tanzania	55	Mix	P	No	Yes	No	Yes	Yes	Meier

*N* number in study, *OM* osteomyelitis, *CHOM* chronic haematogenous osteomyelitis only, *Mix* mixed aetiology of osteomyelitis, *P* paediatric patients only, *P&A* paediatric and adult patients, *XR* plain radiology X-rays used, *LoT* length of treatment reported

The aim of this paper is to assess the application and usefulness of the Beit CURE (BC) classification in clinical practice and to assess the correlation between classification grading and the type and extent of treatment required.

## Methods

### Study methodology

**Fig. 1:**
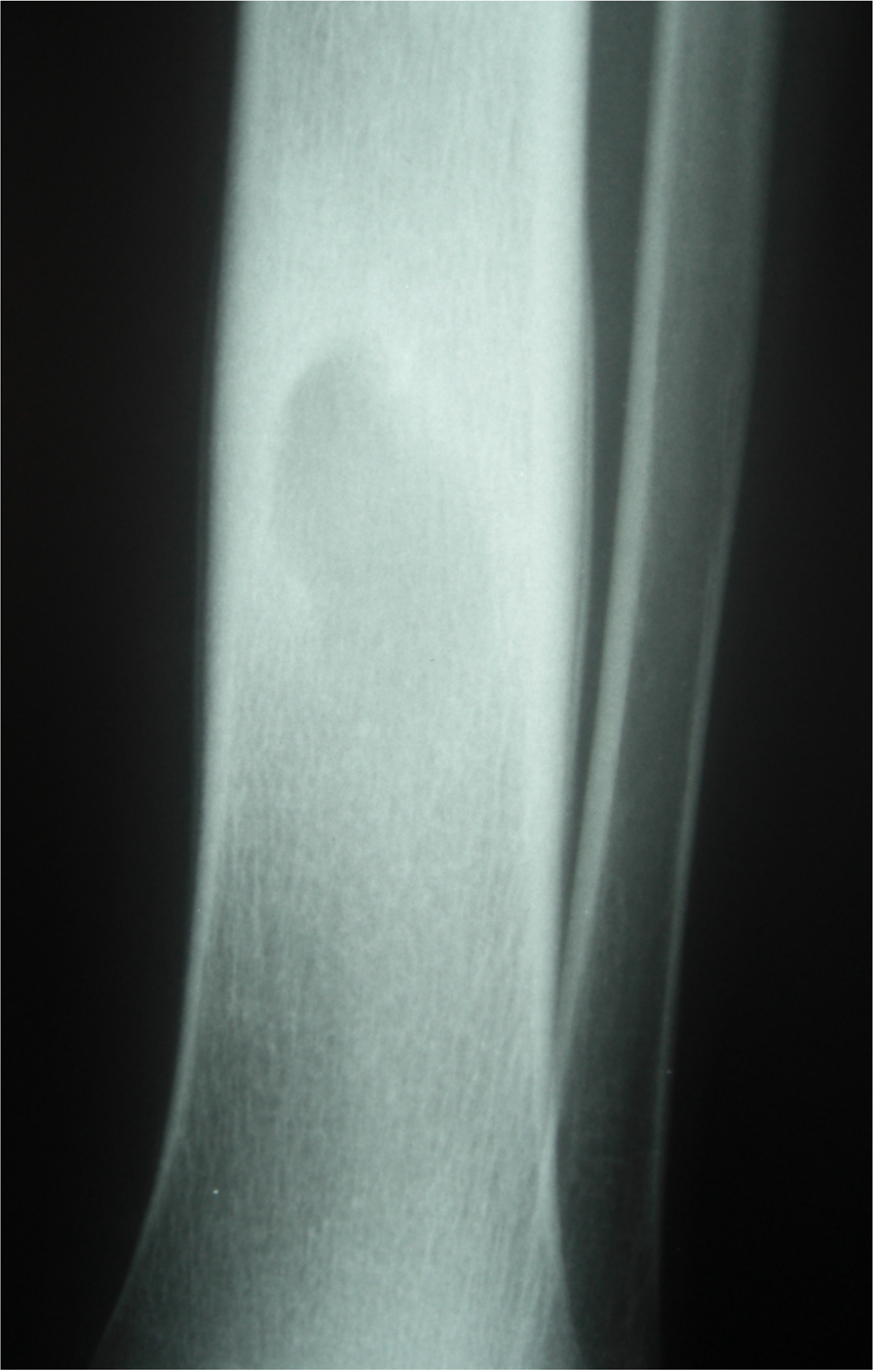
Type A. Brodie’s abscess. No sequestrum. No involucrum

**Fig. 2:**
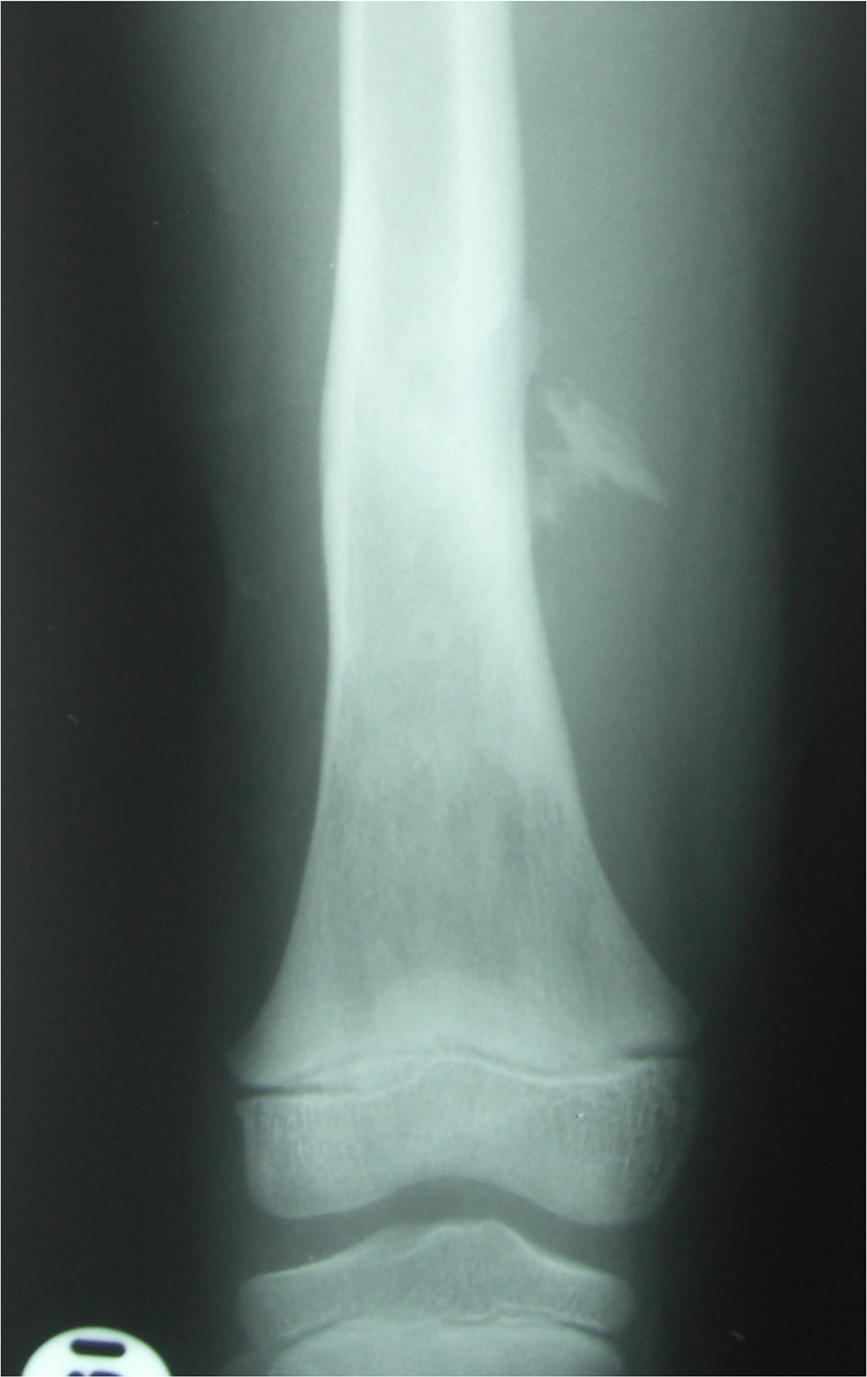
Type B1. Localised cortical sequestrum, nosignificant involucrum

**Fig. 3:**
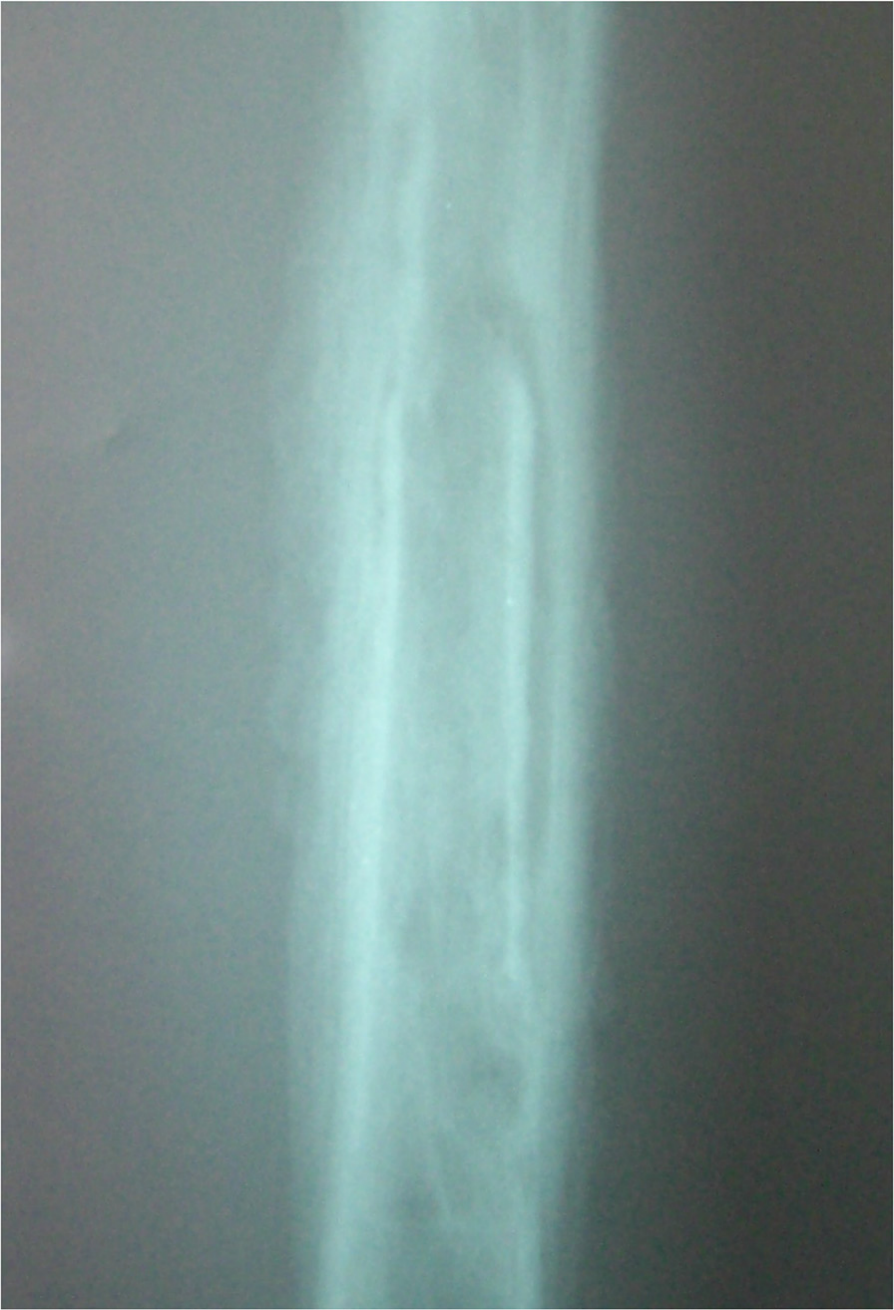
Type B2. Sequestrum present. Structural, normal involucrum

**Fig. 4:**
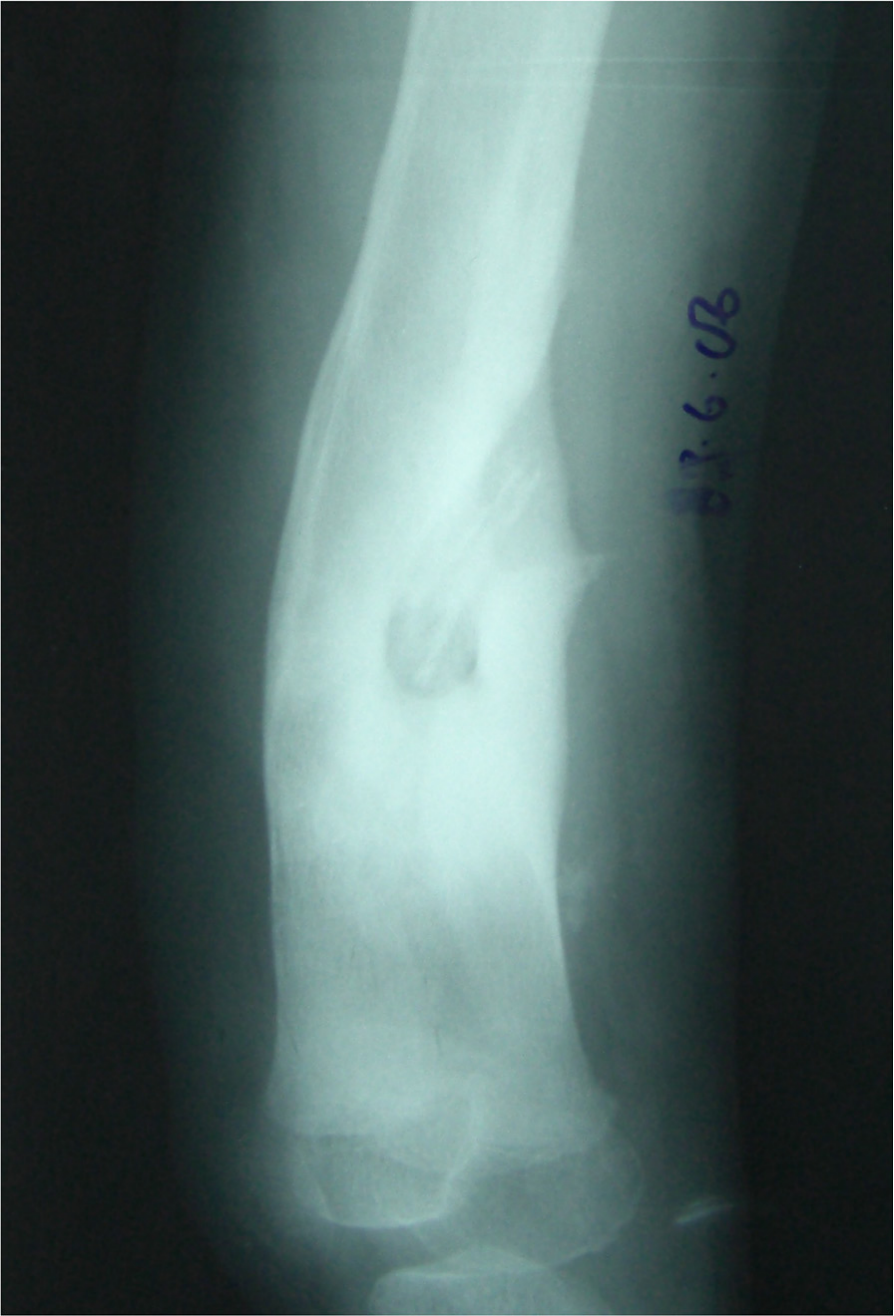
Type B3. Sequestrum present. Structural, sclerotic, expanded involucrum

**Fig. 5:**
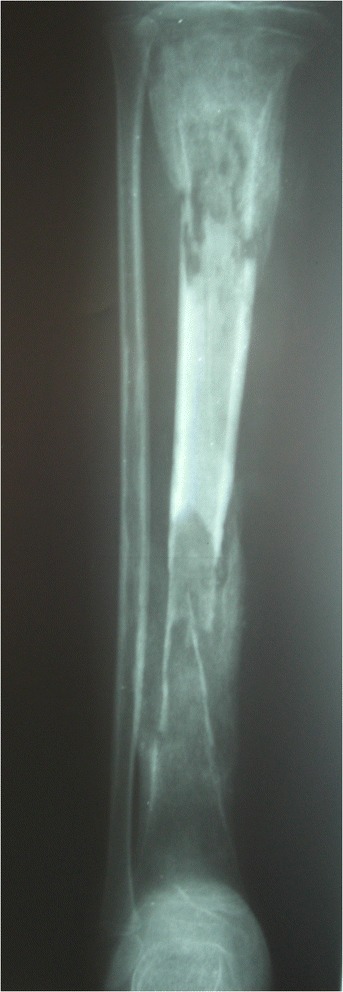
Type B4. Sequestrum present. Non-structural involucrum. Bone defect type

**Fig. 6:**
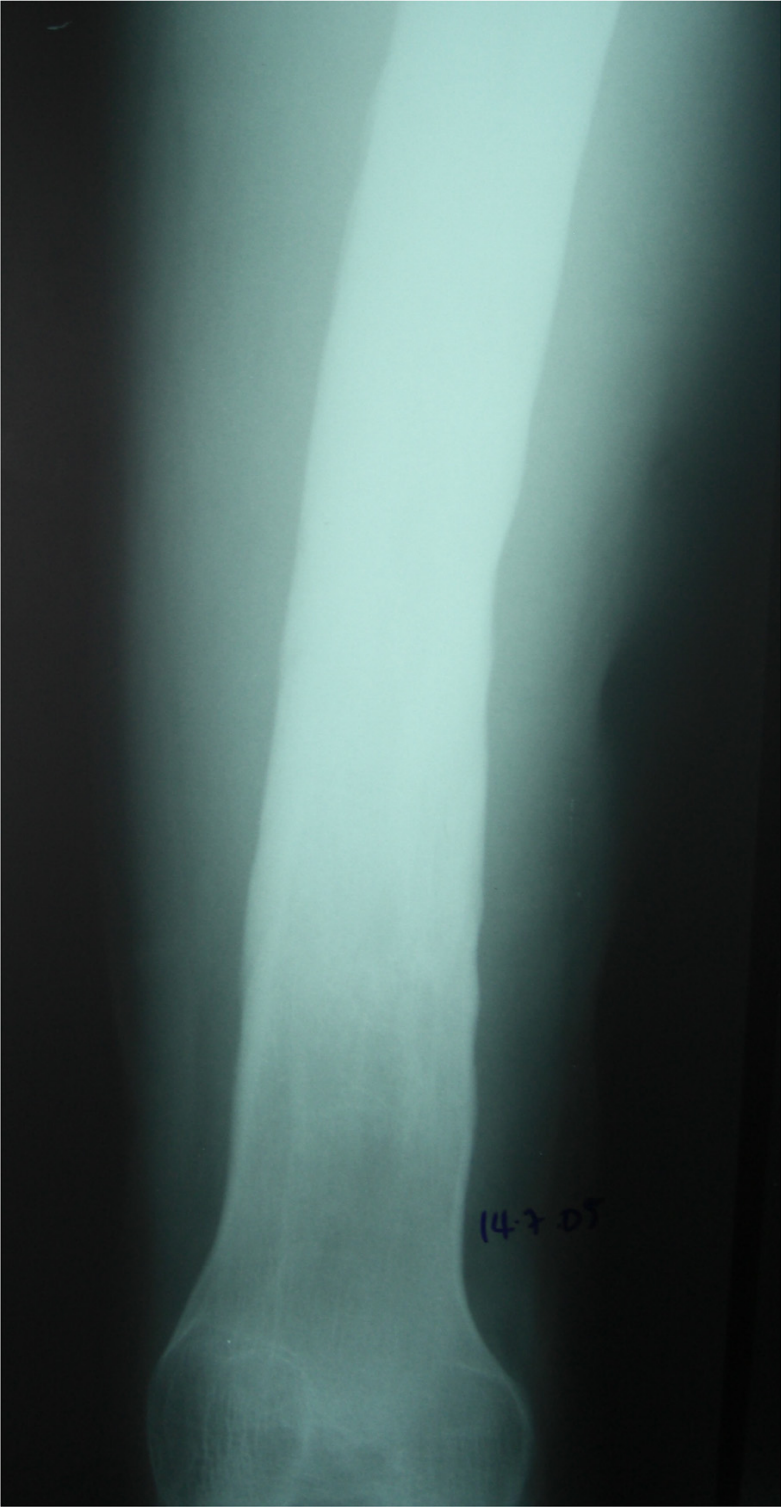
Type C. No sequestrum. Densely sclerotic. Abscess often present

Between January 2003 and December 2007, 167 sequential cases of CCHOM were identified and treated at our institution [1]. Index radiographs of this group of 167 patients were reviewed. These were classified according to the BC classification [32] (Table 2). (Figures 1, 2, 3, 4, 5 and 6 show example radiographs of different types.) The patients aged 18 and under were included, as opposed to under 16 as stated in the original classification criteria. This was done to reflect the delay in skeletal maturity seen in this group of patients, so as to include all skeletally immature patients [33]. Classification was performed by the lead author (AJS). Clarifications of borderline classifications were discussed with the senior author (WJH).

**Table 2:** Summary of the classification criteria of the Beit CURE Classification of Childhood Chronic Haematogenous Osteomyelitis

Classification type	Radiological appearance of bone segment
A	Abscess type, osteolytic area(s), no sequestrum, no involucrum
B1	Peripheral, localised cortical sequestrum; minimal/no involucrum
B2	Sequestrum present; stable, normal-looking cortical involucrum
B3	Sequestrum present; stable, sclerotic involucrum
B4	Sequestrum present; unstable, inadequate involucrum
C	No sequestrum visible on plain X-ray; densely, diffusely sclerotic bone segment; abscess may be present.
Unclassifiable	Inadequate X-ray/disease onset >6-months/previous surgery.

Physeal involvement is indicated by adding a suffix of P for proximal involvement, D for distal involvement and PD for dual physeal involvement

Review of the hospital’s operations database and case notes was performed. Data on age, sex, bone involved, microbiology, number of admissions, total length of hospital stay and number of operations (infection control, reconstruction, other and total) was gathered. Data was collected in December 2010 and therefore represents a minimum 3-year follow-up of the patients in this group.

Ethical approval for the study was granted by the College of Medicine Research and Ethics Committee (COMREC) of the University of Malawi.

### Treatment methodology

All patients had a full clinical assessment (history and physical examination), and over-penetrated radiographs of the affected bone were performed [31]. Haemoglobin and malarial parasite levels were measured pre-operatively. Surgery was performed as indicated according to clinical assessment of the soft tissues and radiological assessment of the bone.

The BC classification was used to guide the type of bony treatment required:

- Type A
- Drilling and curettage of abscess OR conservative treatment with 6 weeks of antibiotics.
- Type B1–B3
- Sequestrectomy and curettage.
- Type B4
- Sequestrectomy and curettage AND stabilisation with plaster, traction or external fixator.
- Reconstruction of bone defect was performed following eradication of infection, as required.
- Type C
- Drainage and curettage of any collection and long-term antibiotics (6 week minimum).

At surgery, pus was drained, sequestrectomy performed (if required) and debridement and lavage carried out. Post-operative radiographs were done to ensure that all sequestra had been removed and to illustrate the post-debridement bony anatomy. Wounds were left to heal by secondary intention, and honey dressings were applied. Soft tissue reconstruction was performed when required. The patients with bone defects were immobilised with plaster, traction or external fixator. If after 3 months insufficient structural involucrum had formed, further reconstruction strategies were employed.

Strategies for surgical treatment of bone defects included free fibula graft, ipsilateral vascularised fibula graft, non-structural bone graft and bone transport. Amputation was used rarely in selected cases. Peri-operative intravenous antibiotics were administered to most patients, with longer courses being prescribed on clinical grounds. The patients were discharged from hospital when wounds were manageable in the community setting, either by a guardian or local health centre. A written discharge summary was given to the patient detailing appropriate follow-up. Follow-up was conducted either at our institution or a local district hospital.

### Statistical methods

Patients’ characteristics in the various BC classification types were assessed using descriptive statistics, and hypothesis testing was done using ANOVA with Bonferroni correction for multiple comparisons. In order to adjust for confounding variables, the association between the various BC classification types and outcomes was assessed using multivariate linear regression models. Statistical significance was set at a *p* value of <0.05. All analysis was done using STATA version 10.0 [34].

## Results and discussion

### Demographics

**Fig. 7:**
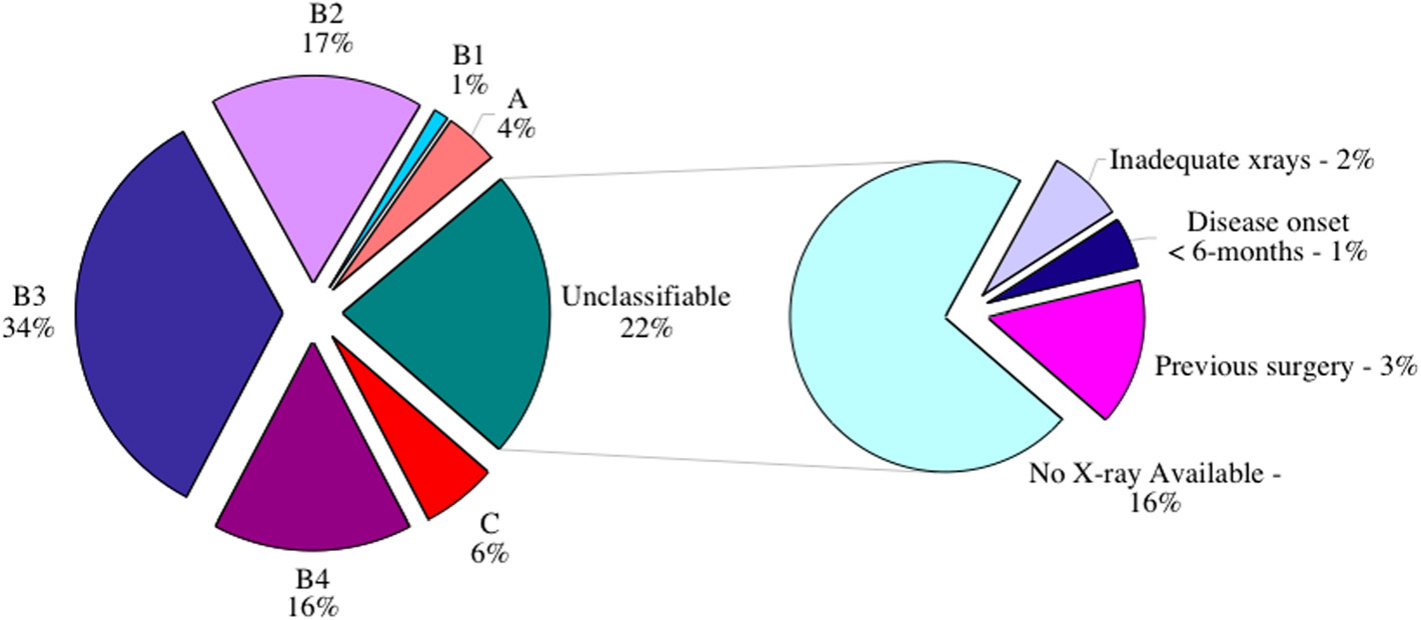
Percentage numbers of cases classified and those that were unclassifiable

**Fig. 8:**
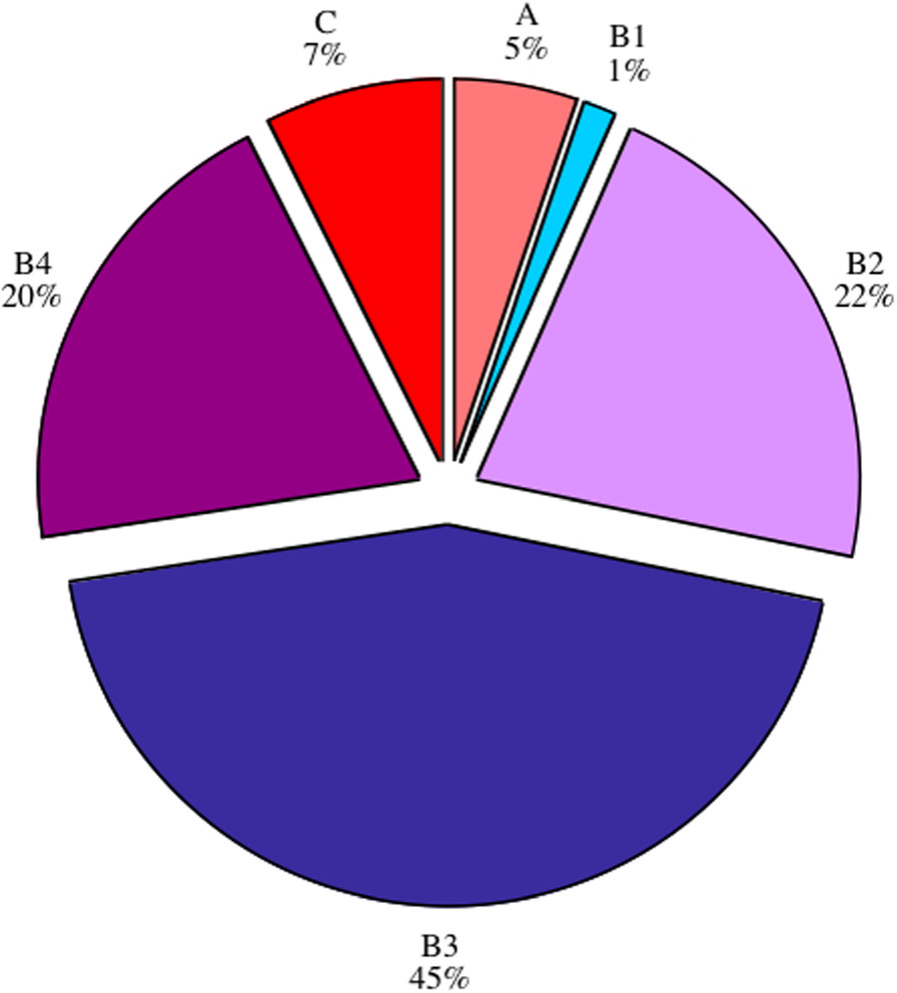
Percentage of each classification type, from a total of 134 bones

Out of 167 patients (102 males, 65 females), 139 patients had available radiographs and notes. Patient ages ranged from 1 to 18 years, with a mean age of 10. One hundred forty-five bones were assessed according to the BC classification (135 cases were monostotic and four polyostotic). One hundred thirty-four bones were classified as type A–C (Fig. 7). Eleven cases were typed ‘Unclassifiable’ due to either of the following: inadequacy of X-rays, previous surgery or <6 months since onset of symptoms (Fig. 8). No significant difference was found in sex and age distribution among the BC classification, as well as among all outcomes.

Throughout statistical analysis of all outcomes, type B1 (*n* = 2) had too few cases to perform any meaningful analysis.

### Classification types

Overall, type A represented 5 %, type B 88 % and type C 7 % of this series of patients. This reflects the fact that most CCHOM produces macroscopic sequestrum, visible on over-penetrated plain radiographs. Brodie’s abscess type (A) and the sclerotic type without sequestrum (C) are relatively less common.

Type B3 was the most common classification (*n* = 59) and B1 the least common (*n* = 2) (Table 3).

**Table 3:** The mean length of stay, mean number of admissions and mean number of procedures

Classification	Number of patients	Length of stay (days)	Admissions	Infection control procedures	Reconstruction procedures	Other procedures	Total number of procedures
A	7	11	1.1	0.9	0	0.3	1.1
B1	2	11	1.0	1.5	0	0	1.5
B2	29	26	1.6	1.9	0	0.2	2.2
B3	59	28	1.4	1.9	0	0.1	2.0
B4	27	87^a^	2.0	2.1	1.9^b^	0.1	4.0^c^
C	10	32	1.6	1.9	0	0.3	2.2

^a^Type B4 has significantly longer inpatient treatment than types A, B2, B3, B4 and C

^b^Type B4 had significantly more reconstruction procedures than all other types

^c^Type B4 had significantly more total number of procedures than all other types

### Bones involved

**Fig. 9:**
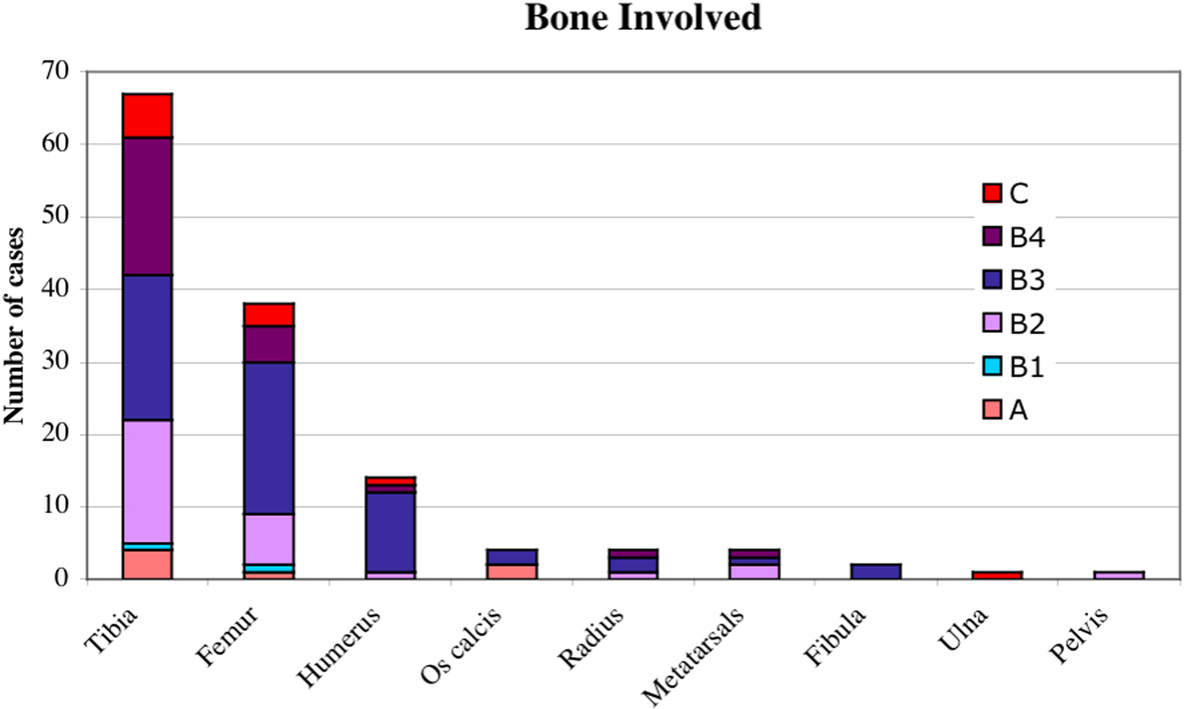
Classification of bones involved, note that the tibia has the greatest number of bone defects (B4)

Nine different bones were involved throughout the series: tibia, femur, humerus, radius, ulna, os calcis, metacarpals, fibula and pelvis. As shown in previous studies, the tibia is the most commonly affected bone [2, 6, 10, 14, 26] followed by the femur and the humerus. The tibia also had the most number of bone defects (B4 classifications) (Fig. 9). This may be due to the tibia’s relatively poor soft tissue cover leaving the periosteum more vulnerable to damage as a result of sepsis, since the viability of the periosteum is the essential factor in the formation of adequate structural involucrum [35, 36].

In all cases, involvement of the physes was found in 37 cases (28 %), with both physes being involved in five of these cases (Table 4).

**Table 4:** Table showing total number of cases and single and dual physeal involvement

Classification type	Total number of cases		Single-physis involvement (P or D)		Dual-physis involvement (PD)	
	Number	%	Number	%	Number	%
A	7	5	1	14	0	0
B1	2	1	0	0	0	0
B2	29	22	8	28	1	4
B3	59	45	16	27	2	4
B4	27	20	6	24	1	4
C	10	7	1	10	1	10
Total	134	100	32	24	5	4

Significantly fewer operations were carried out in non-weight bearing bones (*p* < 0.01) and the foot (*p* < 0.03) than in the tibia.

### Length of stay

Types A and B1 had the shortest length of hospital stay, 11 days each. Types B2, B3 and C had similar lengths of stay, 26, 28 and 32 days, respectively. B4 types had the longest mean length of stay in hospital (87 days), and this was significantly greater than types A, B2 and B3 (*p* < 0.001) and C (*p* = 0.002) (Table 3).

### Number of admissions

Type A and B1 required the least admissions than all the other types as more localised disease is easier to treat and therefore requires fewer admissions. The greater surgical input required in to treat types B and C cases is also reflected in the highest number of admissions (Table 3).

### Infection control procedures

Type A needed the least number of infection control operations (0.9), with some cases treated conservatively with antibiotics alone (Table 3).

Type B cases required sequestrectomy to remove the devitalised bone as part of infection control treatment. Type B1 needed the least number of infection control procedures (mean 1.5) of this group, indicating the relatively low surgical burden of this localised form of the disease. Type B2, B3 and B4 needed a slightly higher number of infection control operations than B1, indicating that the more advanced the disease, the greater the need for multiple procedures.

Type C needed equivalent numbers of infection control procedures as Type B, indicating that although no macroscopic sequestrum was present, repeat surgeries (e.g. drilling, drainage and debridement) were still needed to control infection.

### Reconstruction operations

The number of reconstruction operations in type B4 was significantly greater than all other types (*p* < 0.001) with a mean of 1.9 reconstruction operations being performed (Table 3). This highlights the greater surgical input required in the treatment of bone defects that is not required when bony integrity is not compromised.

### Total number of operations

Types A and B1 had the least total number of operations performed which reflects the less invasive the disease, the less requirement there is for surgery (Table 3).

Similar total numbers of operations were performed in groups B2, B3 and C (2.2, 2.0, 2.2, respectively). Type B4 had a significantly higher total number of operations than all other groups as a result of the reconstruction procedures performed (*p* < 0.001) (Table 3).

### Microbiology

**Fig. 10:**
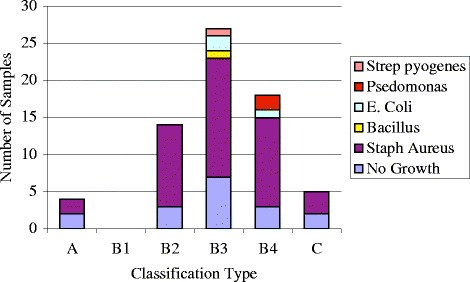
Microbiology of specimens

Microbiology results were available from 68 of the classified cases (Fig. 10). Fifty-one of these cases had positive cultures. *Staphylococcus aureus* was the most common organism found (44 cases). *Bacillus* (*n* = 1), *Escherichia coli* (*n* = 3), *Pseudomonas aeruginosa* (*n* = 2) and *Streptococcus pyogenes* (*n* = 1) were only found in the B3 and B4 groups.

A 75 % (51/68) positive culture rate for the cases in which there were microbiological analyses performed is comparable to other studies [26, 37]; however, only 49 % (58/139) of patients had documented microbiology. This indicates that microbiological management of these patients could be optimised through more rigorous sampling methodology, such as multiple deep tissue samples as opposed to swabs, and a greater frequency of sampling.

## Conclusion

Numerous different classifications for chronic osteomyelitis have been published [2, 3, 13, 28–30]. Waldvogel [27] originally classified osteomyelitis by its aetiology (haematogenous, secondary to focus of infection, chronic), and though descriptive of the origin of disease, it does not aid surgical planning.

The Cierny Mader [28] classification is based on the extent of infection (medullary, superficial, localised, diffuse) and host factors (healthy, immunity compromised, treatment worse than disease). The classification is based on clinical and radiological assessment, as well as host status. This is perhaps the most widely used classification. It represents the pathological progression of osteomyelitis and is useful in planning treatment strategy. However, it is a relatively subjective classification reliant on clinical judgement, interpretation of radiology and assessment of host status. It was also created to classify osteomyelitis in adults and therefore is less transferable to the paediatric population.

Meier [3] classified osteomyelitis as acute, acute with X-ray changes, chronic localised and chronic systemic. It describes acute and chronic disease and does not comment specifically on bony changes.

Lauschke [2] classified paediatric disease from onset of symptoms as early acute (≤3 days), late acute (4–5 days) and chronic (≥6 days). This narrow time-dependant classification does not recognise more extensive disease progression and is therefore not applicable to the majority of CCHOM seen in clinical practice.

Solagberu’s [13] classification is based on the progression of disease, ranging from ‘pre-invasion’ to ‘compound chronic’. The subjectivity of the grading of this classification makes reproducibility difficult.

The BC classification [32] is the first classification devised solely for the use of CCHOM. It is also unique in the explicit use of the plain radiograph in its classification. Furthermore, it has been assessed for intra- and inter-observability, a vital aspect of a reliable, reproducible, classification system. No other classifications have undergone this process. The BC classification comments on the both sequestrum and involucrum and therefore makes it a useful tool in the planning of treatment. In CCHOM, it is the bone infection that drives the course of the disease. The accurate identification of the bony pathology that the BC classification allows therefore plays a central role in guiding treatment.

This study shows that the BC classification is prognostic of the type and extent of bony treatment required.

Types A and B1 both required the least surgical input overall and had the shortest inpatient stay. Types B2, 3, 4 and C all required similar number of infection control procedures. However, of these, type B4 required the most admissions, operations and inpatient treatment as a result of the reconstructive procedures needed to treat bone defects present.

This study has several weaknesses. It is a retrospective study and therefore is subject to a degree of variation in assessment and treatment of patients that can occur without the strict protocols of a prospective design. The data describes the total amount and type of inpatient treatment; however, it does not distinguish between the initial treatment episode and subsequent treatment of recurrence, so no firm conclusions can be made as to rate of recurrence.

Though inpatient hospital notes were accurate and well written, the outpatient hospital attendance was recorded in a separate patient-held health book to allow continuation of care in different hospitals. This meant that clinical outcome was difficult to determine. The accurate documentation of follow-up is variable. Inconsistent follow-up is highlighted in another recent study that had a 47 % rate of patients who failed to attend follow-up [14].

Chronic haematogenous osteomyelitis in children is a difficult disease to treat, largely affecting children in the lesser-developed nations. There are no worldwide figures on incidence or prevalence, and comparable research is lacking. Though largely occurring in tropical countries, CCHOM is not listed in the World Health Organisation Report on Neglected Tropical Diseases [38] and does not attract the international profile nor funding that other eradication programmes for more definable disease are receiving.

The Beit CURE classification is simple, reliable, reproducible, relevant and prognostic classification for CCHOM.

In all previous studies, plain radiographs were used (Table 1) and therefore would have been suitable for use of the BC classification.

The use of plain radiographs, widely available throughout the developing world, makes it reproducible and objective. The classification grading from A to C aids the planning of treatment and indicates the likely number and type of procedures required, as well as the total length of inpatient treatment.

Correlating of the classification grade with length of stay and number and type of procedures required could also help strategic health planning and resource allocation.

The adoption of the BC classification in other treating centres will assist greatly in much-needed comparative studies and research to improve on care strategies for this prevalent and debilitating disease of the developing world. Prospective, well-resourced studies are the next step in progressing the treatment for this disease that affects so many of the world’s most vulnerable children.
